# Long-Term Limitation Effects of Se(VI), Zn(II), and Ni(II) on Start-Up of the Anammox Process Using Gel Carrier

**DOI:** 10.3389/fbioe.2022.851617

**Published:** 2022-03-04

**Authors:** Kazuichi Isaka, Daichi Sugawara, Hiroshi Yamazaki, Yuya Kimura, Toshifumi Osaka, Satoshi Tsuneda

**Affiliations:** ^1^ Department of Applied Chemistry, Toyo University, Saitama, Japan; ^2^ Department of Civil and Environmental Engineering, Toyo University, Saitama, Japan; ^3^ Water and Environment Business Unit, Hitachi, Ltd., Tokyo, Japan; ^4^ Department of Microbiology and Immunology, Tokyo Women’s Medical University, Tokyo, Japan; ^5^ Department of Life Science and Medical Bioscience, Waseda University, Tokyo, Japan

**Keywords:** anammox, trace element, heavy metal, limitation, inhibition

## Abstract

Anaerobic ammonia oxidation (anammox) bacteria are inhibited by heavy metals at high concentrations but require trace amounts of some heavy-metal elements for growth and activity maintenance. The present study evaluates the long-term limitation effects of Se(VI), Zn(II), and Ni(II) on the start-up period of an anammox reactor. To strictly limit the levels of heavy metals in the reactor, all tests used ultrapure water as the influent synthetic wastewater and all reactors were installed in a clean booth. The anammox biomass was maintained through the gel entrapment technique. In the absence of Se(VI) and Ni(II), the anammox reactor start-up was 18.9 kg-N (m^3^-carrier d)^−1^ (nitrogen conversion rate (NCR) per gel carriers), indicating that Se(VI) and Ni(II) are not required or need not be continuously added to maintain the anammox process. Under Zn(II) limitation, the anammox process failed to start-up and the NCR tended to decrease rapidly. After readdition of 0.005 mg L^−1^ of Zn(II), the NCR did not decline further and instead partially recovered at a very slow rate. The NCR was completely recovered after adding 0.020 mg L^−1^ of Zn(II). These results reveal that Zn(II) limitation seriously affects the start-up of the anammox process while Se(VI) and Ni(II) are not required or need not be continuously added to the anammox process.

## Introduction

Anaerobic ammonium oxidation (anammox) is a novel biochemical reaction and anammox processes are currently employed in cost-effective wastewater treatments (Mulder et al., 1995; Strous et al., 1999; Strous et al., 2006; van der Star et al., 2007). The anammox process is mainly used in the digester supernatant treatment of municipal wastewater (van der Star et al., 2007; Lackner et al., 2014). Additionally, it is applied to treat industrial wastewaters such as semiconductor wastewater and ammonia-plant wastewater (Tokutomi et al., 2011; Isaka et al., 2017). The anammox bacteria require trace amounts of heavy metals for metabolism and growth. Such trace elements (TEs) mainly act as coenzymes and the cofactors of enzymes (Fermoso et al., 2009; Glass and Orphan, 2012). Anammox activity is reportedly increased by adding heavy metals. For instance, Zhang et al. (2019) reported that a long-term exposure to 1 mg L^−1^ of Fe(II) enhanced the anammox activity, and Sindhu et al. (2021) reported an increase in the nitrogen removal performance of a sequencing batch reactor (SBR) after adding 55.8 mg L^−1^ of Fe(II). The heavy-metal contents of industrial wastewater are expected to be insufficient for microorganism growth because factories use pure water in the production lines.

Conversely, high concentrations of heavy metals greatly reduce the anammox activity. Gutwínski et al. (2021) examined the long-term effect of heavy metals on the anammox process using an SBR and found that heavy-metal mixtures of Zn(II), Cu(II), and Ni(II) at high concentrations inhibited the anammox activity. They suggested that Zn(II) (0.8 mg L^−1^) was the main inhibitor. The half maximal inhibitory concentration (IC_50_) of Ni(II) was determined as 14.6 mg L^−1^ in a short-term batch test (Xu et al., 2017).

These results confirm that although some types of heavy metals are essential for the growth of anammox bacteria, they inhibit the anammox activity at high concentrations. Therefore, to maintain a stable anammox process, a detailed investigation is required regarding the types of heavy metals essential for anammox bacteria growth and the metals’ minimum concentrations (van de Star et al., 2007). suggested nine heavy metals—Fe(II), Zn(II), Mn(II), Cu(II), Ni(II), Mo(VI), Co(II), B(III), and Se(VI)—that are essential for cultivating anammox bacteria. Stable cultivation in the presence of these nine heavy metals has been fundamental to the development of current anammox research. However, the necessity of these elements for the growth of anammox bacteria and stable nitrogen removal performance has not been reported. If the essential TEs are insufficiently provided in the wastewater, they should be added as chemicals. Clarifying which TEs are actually required would generalize the anammox process to various types of industrial wastewater treatments. Furthermore, understanding the impact of each heavy metal on the nitrogen removal performance is extremely important for understanding the metabolism related to the anammox reaction. The necessity of Se(VI) in the anammox process is especially relevant, as selenium is highly toxic and its usage is strictly restricted. In addition, the necessity of Ni(II) and Zn (II) for the anammox process should be investigated because the inhibitory effect of high concentrations of Ni(II) and Zn(II) on anammox bacteria has been reported.

Therefore, herein, we investigated the limiting effects of Se(VI), Zn(II), and Ni(II) on the start-up of the anammox process. When assessing the need for TEs in anammox reactions, several technical challenges must be overcome. As heavy metals are accumulated in the anammox cells during precultivation, it is difficult to determine their requirement in short-term tests such as batch tests. Meanwhile, long-term tests require a continuous supply of a nitrogen medium, risking the washout of anammox biomass from the test reactor. To resolve these issues, we conducted continuous feeding tests and immobilized the cells in a polyethylene glycol (PEG) gel carrier (Isaka et al., 2007). Although several types of reactors for anammox processes have been reported, including the SBR, up-flow anaerobic sludge blanket (UASB), and moving-bed bioreactor (MBBR) (Strous et al., 1998; Strous et al., 1999; Sliekers et al., 2003; Furukawa et al., 2006; Dimitrova et al., 2020; Li et al., 2021; Peng et al., 2022). The gel entrapment technique was used to prevent the washout of anammox biomass from the reactor (Isaka et al., 2007). Moreover, ultrapure water was used for all wastewater tests to avoid contamination by heavy metals and all test systems were installed in a clean booth equipped with a fan filter unit (FFU). The limiting effects of Se(VI), Zn(II), and Ni(II) on the start-up of the anammox process were evaluated individually. To our knowledge, this is the first study to report that Zn(II) limitation seriously affects the start-up of the anammox process while Se(VI) and Ni(II) do not.

## Materials and Methods

### Seed Sludge

For the immobilization of anammox gel carriers, the anammox bacteria in the seed sludge were enriched from sewage sludge and grown in a 50-L fixed-bed reactor filled with porous polyester nonwoven fabric carriers at 35°C (Fujii et al., 2002). The reactor was continuously fed with synthetic wastewater at an approximately constant loading rate of 0.8 kg-N (m^3^d)^−1^. The synthetic wastewater was prepared using tap water and all heavy metals except Se(VI) were added at the levels reported in a previous study (van de Star et al., 2007).

### Gel Carrier

The anammox sludge was entrapped in a PEG gel carrier as described by Isaka et al. (2007). The polymerized gel carrier was cut into 3-mm-sided cubes. The gel carrier contained 10% (w/v) PEG prepolymer, 0.5% (w/v) *N*,*N*,*N*′,*N*′-tetramethylethylenediamine (as promoter), 0.25% (w/v) potassium persulfate (as initiator), and 1.0% (w/v) anammox bacteria-enriched sludge. To distribute gel carriers with the same activity to each system, all gel carriers were precultured in a large reactor until a moderate anammox activity (∼4 kg-N (m^3^-carrier d)^−1^) was obtained.

### Synthetic Wastewater

The basic synthetic wastewater for the continuous feeding tests contained the following compounds: (NH_4_)_2_SO_4_ (169 mg-N L^−1^), NaNO_2_ (210 mg-N L^−1^), NaHCO_3_ (500 mg L^−1^), MgSO_4_·7H_2_O (300 mg L^−1^), CaCl_2_·2H_2_O (180 mg L^−1^), and KH_2_PO_4_ (27.2 mg L^−1^). The concentrations of ammonium and nitrite were changed during the start-up of the reactor to avoid nitrite inhibition. The basic TE solution contained ethylenediaminetatraacetic acid (10.6 mg L^−1^), MnCl_2_·4H_2_O (0.99 mg L^−1^), ZnSO_4_·7H_2_O (0.43 mg L^−1^), FeSO_4_·7H_2_O (5 mg L^−1^), CuSO_4_·5H_2_O (0.25 mg L^−1^), NiCl_2_·6H_2_O (0.19 mg L^−1^), Na_2_MoO_4_·2H_2_O (0.22 mg L^−1^), CoCl_2_·6H_2_O (0.24 mg L^−1^), and H_3_BO_3_ (0.014 mg L^−1^) (van der Star et al., 2007). As Se(VI) is toxic and the anammox activity is unaffected by the long-term absence of Se(VI) in the seed-sludge cultivation (data not shown), Se(VI) was excluded from this experiment. Table 1 shows the TE concentrations used in each test. Continuous tests were conducted in three separate reactors: Run1 (Se limitation), Run2 (Zn limitation), and Run3 (Ni limitation).

**TABLE 1:** Heavy-metal concentrations in the limitation tests.

Element	Additive concentration (mg L^−1^)			
	—	Run1	Run2	Run3
	Precultivation	Se limitation (control)	Zn limitation	Ni limitation
Zn	0.098	0.098	0	0.098
Ni	0.047	0.047	0.047	0
Co	0.059	0.059	0.059	0.059
Mn	0.275	0.275	0.275	0.275
Fe	1.004	1.004	1.004	1.004
Cu	0.064	0.064	0.064	0.064
Mo	0.087	0.087	0.087	0.087
B	0.002	0.002	0.002	0.002
Se	0	0	0	0

To control the concentration of each heavy metals, synthetic wastewater and the TE solutions were prepared using ultrapure water (FP-0120*α*-M00, ORGANO). As dissolved oxygen (DO) exerts an inhibitory effect on anammox activity, the DO concentration of the synthetic medium was reduced to ≤0.5 mg L^−1^ by sparging with N_2_ gas.

### Reactor and Experimental Conditions

**FIGURE 1:**
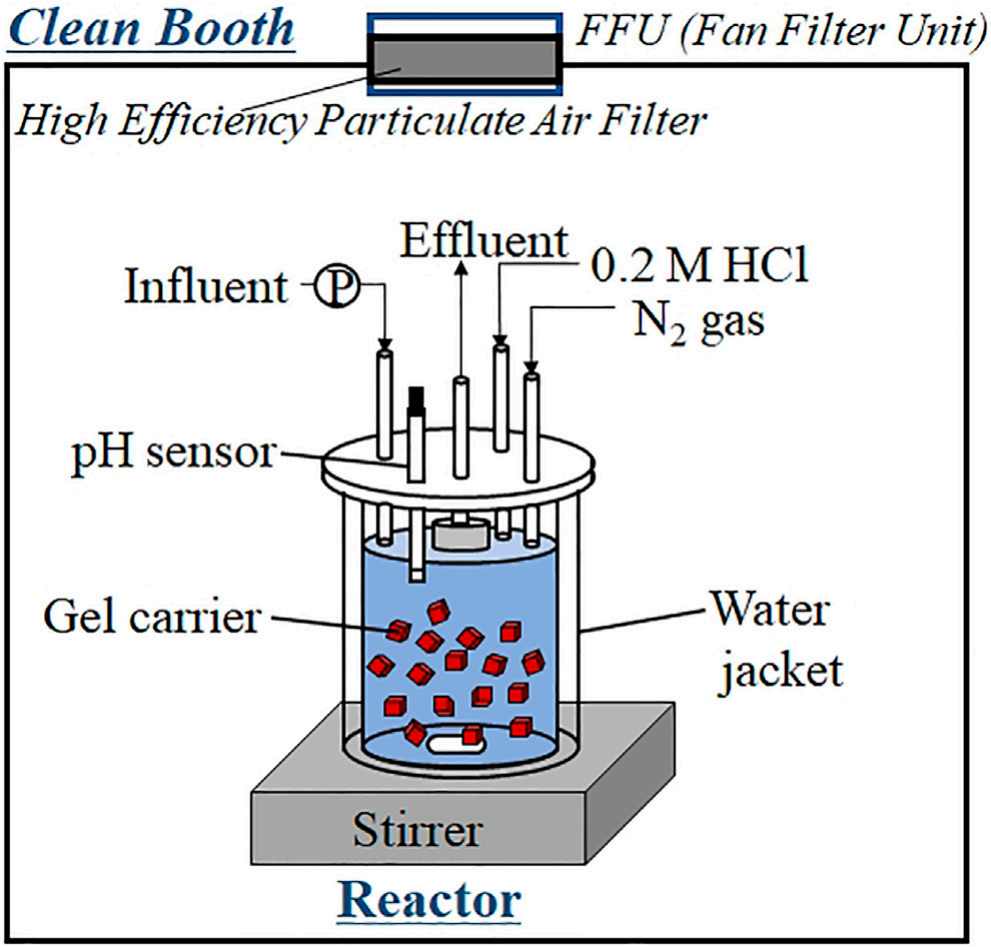
Schematic of the continuous feeding reactor used in the trace-element limitation test. The reactor was set in a clean booth equipped with a fan filter unit.

Continuous feeding tests were performed in 500-ml cylindrical reactors (Figure 1) packed with 50 ml of gel carrier (packing ratio = 10%). The influence of each heavy metal was evaluated in an independent test reactor. Each reactor was enclosed in a water jacket that maintained its temperature at 35°C. The gel carriers were agitated continuously by a stirrer. The pH in the reactor was monitored and adjusted to 7.6 using 0.2-M hydrochloric acid. N_2_ gas was continuously injected into the head-space of the reactor to avoid oxygen contamination and maintain anaerobic conditions inside the reactor. All reactors were installed inside a clean booth with an FFU containing a highly efficient air-particle filter (PS01-AD, As one, Tokyo). This setup avoided heavy-metal contamination of the reactors and protected the effluent samples from airborne dust. The carrier was precultured in a large reactor to confirm the anammox activity of the gel carriers. The shape of the precultivation reactor was identical to that of the continuous feeding reactor, as shown in Figure 1, but the precultivation reactor had larger volume (3.0 L). After confirming sufficient anammox activity (a nitrogen conversion rate (NCR) per gel volume of 4 kg-N (m^3^-carrier d)^−1^), the carriers were dispensed into the 0.5-L test reactors (Figure 1) for conducting the heavy-metal limitation tests. The heavy-metal limitation test was initiated several days after start-up, allowing sufficient increase in the anammox activity. The hydraulic retention time (HRT) was set from 4.4 to 8.3 h (depending on the increase in anammox activity) to prevent the accumulation of high concentrations of nitrite.

### Chemical Analysis

Immediately after sampling, the influent and effluent samples were passed through a disposable membrane filter unit using a 0.45-μm filter (DISMIC, ADVANTEC, Tokyo, Japan). The nitrite and nitrate concentrations were determined using ion chromatography (ICS-1000, Thermo Fisher Scientific, United States) based on the Japanese Industrial Standards (JIS) K 0127 standard (Jis 2017). The ammonium level was measured using the coulometric titration method (AT-2000, Central Kagaku Corp, Japan). Heavy-metal concentrations were measured via inductively coupled plasma mass spectrometry (ICP–MS) (iCAP Q, Thermo Fisher Scientific, United States) based on the JIS K 0133 standard (Jis 2007).

### Characterization of the Microbial Communities

For DNA extraction from the anammox cells, approximately 100 mg of the gel carrier was homogenized in microtubes. The total DNA was extracted using the ISOPLANT kit (Nippon Gene Inc., Toyama, Japan). The V7–V8 hypervariable regions of the 16S rRNA gene were amplified using 1055F/1392R primers (Ferris et al., 1996) and Ion Xpress Barcode Adaptor sequences. The amplicon samples obtained through the polymerase chain reaction were purified on E-Gel SizeSelect II agarose gels (Thermo Fisher Scientific, Waltham, MA, United States). Following the manufacturer’s instructions, a template sample was prepared using the Ion OneTouch 2 system (Thermo Fisher Scientific, Waltham, MA, United States) and an Ion PGM Hi-Q View OT2 kit (Thermo Fisher Scientific, Waltham, MA, United States). Sequencing was performed on an Ion Torrent PGM system (Thermo Fisher Scientific) using an Ion PGM Hi-Q View sequencing kit and an Ion 318 Chip kit v2 BC (Thermo Fisher Scientific). The readings of the sequencing were trimmed to remove the adaptor and barcode sequences. High-quality filtering was performed on a CLC genomics workbench (Qiagen, Aarhus, Germany), setting a minimum quality score of 0.05 and a sequencing length of 300–400 bp. The obtained sequence data were processed using QIIME 1.7.0 (Caporaso et al., 2010).

## Results

### Start-up of anammox reactor under Se(VI) limitation (Run 1)

**FIGURE 2:**
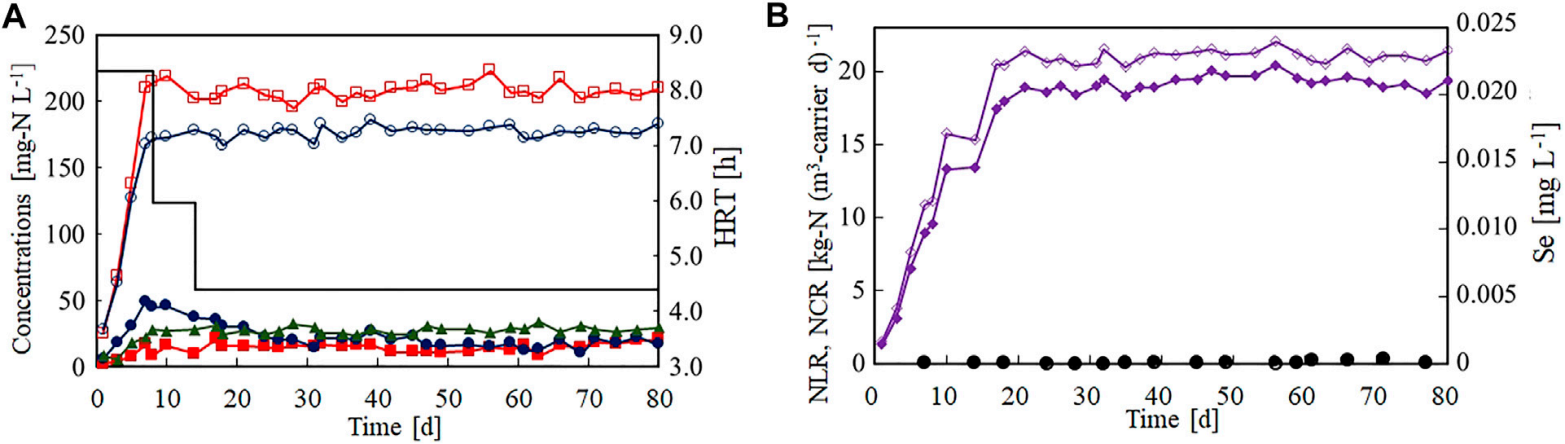
Start-up of the anammox reactor packed with polyethylene glycol gel carrier in the Se(VI)-limitation experiment. **(A)** Time courses of influent ammonium (open circles), influent nitrite (open squares), effluent ammonium (filled circles), effluent nitrite (filled squares), and effluent nitrate (filled triangles). The bar graph shows the hydraulic retention time on each day. **(B)** Time courses of nitrogen loading (open diamonds) and nitrogen conversion rate (filled diamonds).

Figure 2 shows the nitrogen removal performance of the anammox reactor in the Se(VI)-limitation experiment (Run 1). Panel (A) of this figure presents the time courses of the nitrogen and ammonium concentrations in the influent and effluent. The influent ammonium and nitrite concentrations increased in the first 7 days, but effluent both concentrations remained lower than 50 mg-N L^−1^. The concentration of effluent nitrate produced by the anammox reaction increased with an increase in influent nitrogen concentration. The nitrogen removal performance was stable even after decreasing the HRT to 6.0 and 4.4 h on days 10 and 14, respectively. Figure 2B shows the time courses of the nitrogen loading rate (NLR) and NCR per volume of gel carriers. The NCR rapidly increased with increasing NLR and reached 18.9 kg-N (m^3^-carrier d)^−1^ on day 21. The average NLR and NCR were 21.1 and 19.2 kg-N (m^3^-carrier d)^−1^, respectively. The influent and effluent Se(VI) concentrations are also plotted in Figure 2B. Clearly, the influent Se(VI) concentration remained lower than 0.001 mg L^−1^ during the Se(VI)-limitation test.

Selenium is known as an essential TE for humans but is strictly regulated in Japan because it a toxic substrate. In our laboratory, seed anammox sludge has been cultivated for several years in the absence of influent Se(VI) with no effect on the anammox activity (data not shown). Therefore, we concluded that Se(VI) is not required for the anammox process and the subsequent heavy-metal limitation tests were conducted without Se(VI).

### Effect of Zn(II) Limitation (Run 2)

**FIGURE 3:**
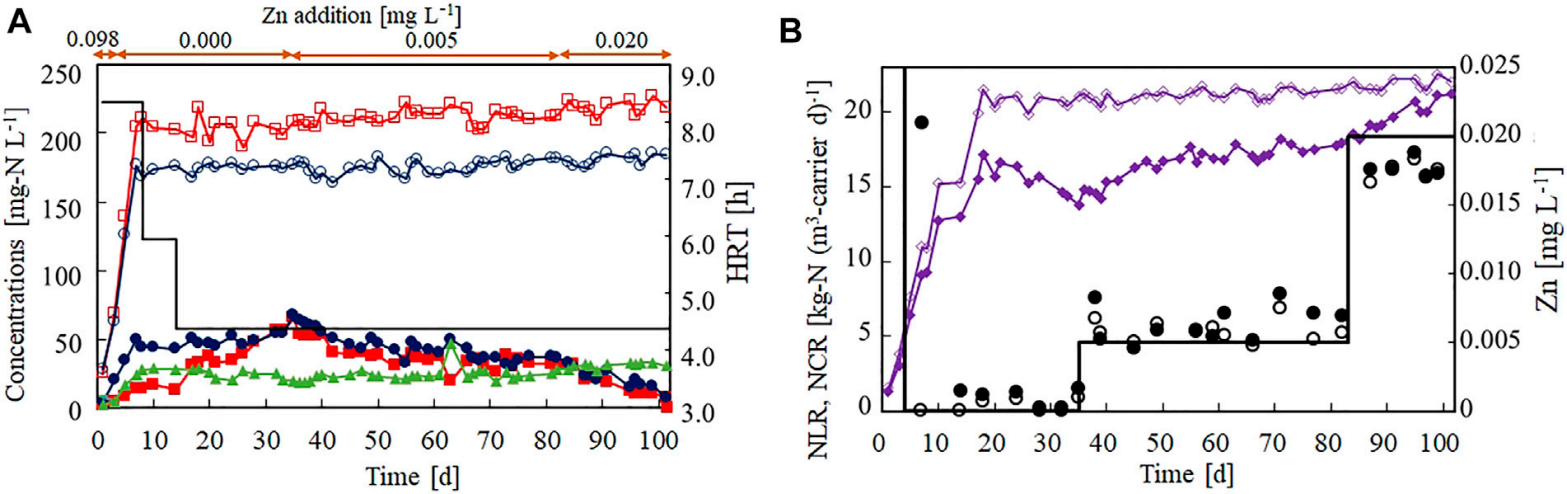
Effect of Zn(II) concentration on the nitrogen removal performance of the anammox reactor. **(A)** Time courses (in days) of influent ammonium (open circles), influent nitrite (open squares), effluent ammonium (filled circles), effluent nitrite (filled squares), and effluent nitrate (filled triangles). The bar graph shows the hydraulic retention time on each day. **(B)** Time courses of nitrogen loading (open diamonds) and nitrogen conversion rate (filled diamonds). The bar graph shows the set value of the influent Zn(II) on each day. The open and closed circles indicate the influent and effluent Zn concentrations, respectively.

Figure 3 shows the effect of Zn(II) limitation on the nitrogen removal performance of the anammox reactor. Zn(II) was removed from the influent on day 4, but the anammox activity was not lost immediately (Figure 3A). The NCR increased to 17.2 kg-N (m^3^-carrier d)^−1^ on day 18 (Figure 3B) and gradually decreased to 13.8 kg-N (m^3^-carrier d)^−1^ by day 35. These results indicated that the anammox process was strongly affected by Zn(II) limitation. During the Zn(II)-limitation test, the influent Zn concentration was maintained below 0.001 mg L^−1^ (Figure 3B).

It has been reported that nitrite inhibits anammox bacteria. Thus, an increase in the effluent nitrite concentration to 64 mg-N L^−1^ (on day 35) by Zn limitation could inhibit the anammox activity. Therefore, the addition of Zn (II) was re-started on day 35 at a concentration of 0.005 mg L^−1^. Consequently, the NCR ceased decreasing and started increasing at a slow rate. However, the anammox activity was not completely recovered after ∼50 days of operation. After increasing the influent Zn(II) concentration to 0.020 mg L^−1^, the recovery rate of the NCR quickly increased and reached 21.2 kg-N (m^3^-carrier d)^−1^ on day 101.

Preliminarily to Run2, a similar Zn(II)-limitation test was performed without the ICP–MS measurements. The NCR reached 19.5 kg-N (m^3^-carrier d)^−1^ on day 21, but both the nitrite and ammonium concentrations in the effluent slowly increased, indicating that Zn(II) limitation deactivated the anammox bacteria (Supplementary Figure S1). Similarly, it was confirmed that the anammox activity ceased declining after adding 0.005 mg L^−1^ of Zn(II) and was recovered by adding 0.020 mg L^−1^ of Zn(II).

These results confirm that Zn(II) is required for maintaining anammox activity. More specifically, Zn(II) must be supplied at a concentration of 0.005 mg L^−1^ to maintain the anammox activity and at 0.020 mg L^−1^ to recover the anammox activity after deactivation by Zn(II) limitation. The recovery level of Zn(II) (0.020 mg L^−1^) is approximately one-fifth of that reported in a previous study (0.098 mg L^−1^; (van der Star et al., 2007).

### Effect of Ni(II) Limitation (Run3)

**FIGURE 4:**
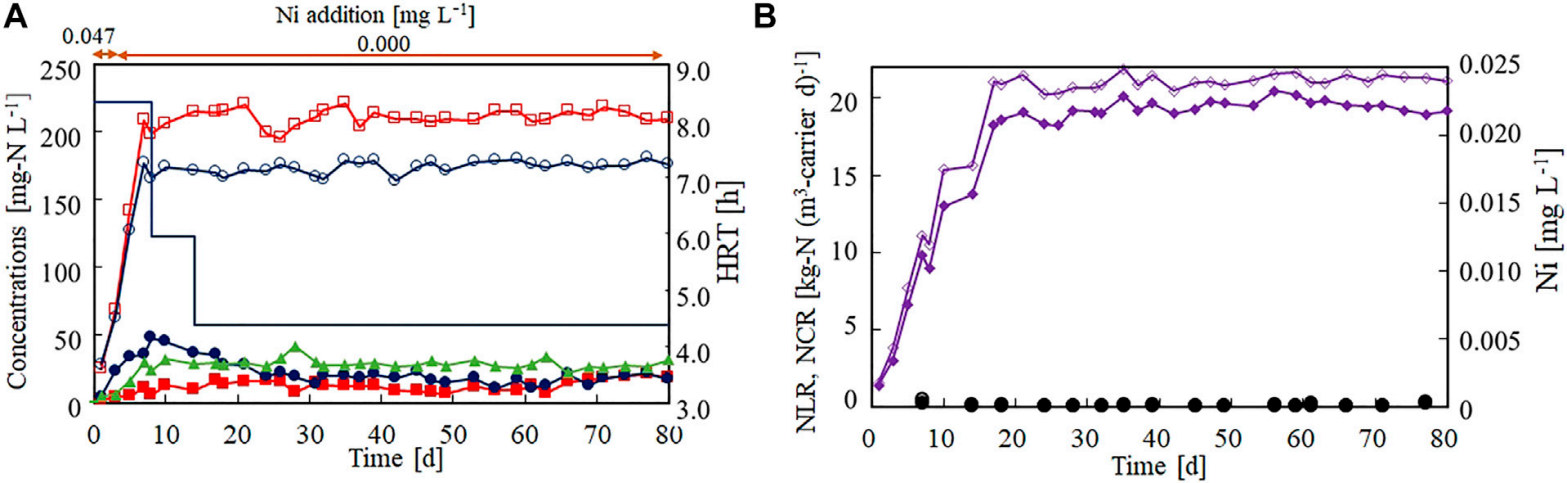
Effect of Ni(II)-limitation on the start-up of the anammox reactor packed with gel carrier. **(A)** Time courses of influent ammonium (open circles), influent nitrite (open squares), effluent ammonium (filled circles), effluent nitrite (filled squares), and effluent nitrate (filled triangles). The bar graph shows the hydraulic retention time on each day. **(B)** Time courses of nitrogen loading (open diamonds) and nitrogen conversion rate (filled diamonds). The open and closed circles indicate the influent and effluent Ni concentrations, respectively.

The effect of Ni(II) limitation on the anammox activity was evaluated in a continuous feeding test. The nitrogen removal performance remained stable for more than 2 months under the Ni(II)-limited environment (Figure 4A). The NCR reached 19.0 kg-N (m^3^-carrier d)^−1^ on day 21 and averaged as 19.4 kg-N (m^3^-carrier d)^−1^ thereafter (Figure 4B). The activity was not noticeably changed from that of Run1 with Ni(II) addition. Both the influent and effluent Ni concentrations remained lower than 0.001 mg L^−1^, revealing that Ni(II) was strictly limited throughout this continuous feeding test. From these results, it was inferred that Ni(II) is not required or need not be continuously added to the anammox process.

### Effect of Zn(II) Limitation on the Microbial Community

**FIGURE 5:**
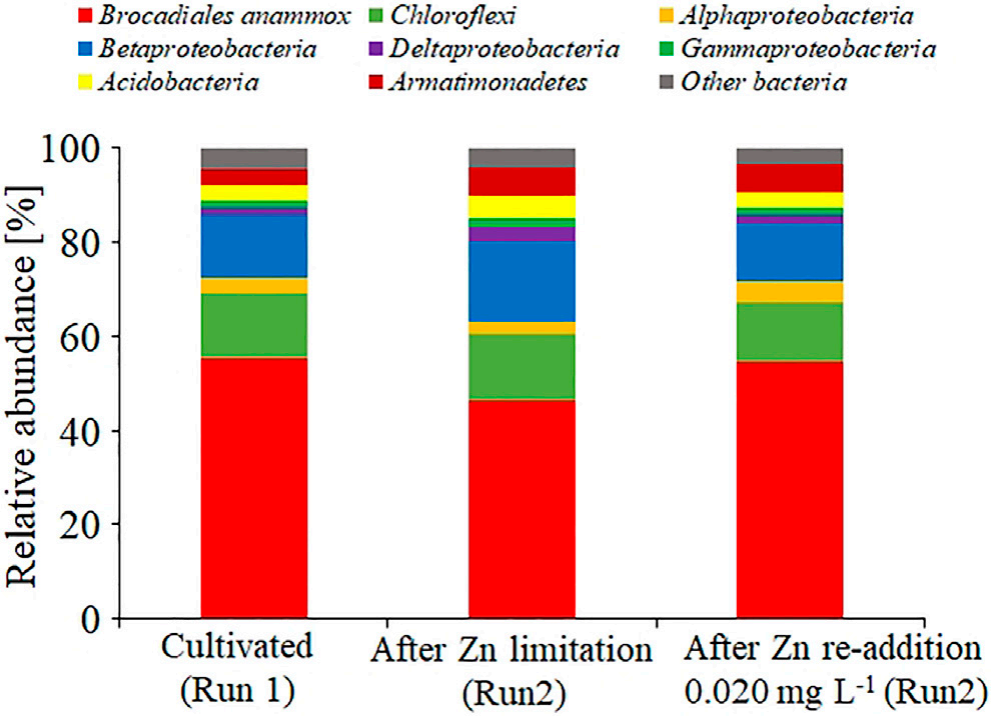
Bacterial community structure in the gel carriers based on a meta 16S rRNA gene sequencing analysis. Results were obtained after cultivating without Zn(II) limitation (Run 1), after Zn(II) limitation (Run 2), and after adding 0.020 mg L^−1^ of Zn(II) to the Zn(II)-limited culture.

The microbial community in gel carriers was analyzed via 16S rRNA gene amplicon sequencing (Figure 5). Figure 5 shows the dominance of anammox bacteria affiliated within the order *Brocadiales* in gel carriers cultivated under the Se(VI)- or Zn(II)-limitation condition (Run 1, 55.3%; Run 2, 46.4%). More than 90% of the anammox bacteria in each reactor had 100% similarity with *Candidatus* Kuenenia stuttgartiensis. Moreover, in Run 2, the readdition of Zn(II) promoted a slight increase in the anammox bacterial population from 46.4 to 54.5%.

## Discussion

Heavy metals are essential TEs for anammox bacteria but are known to inhibit the anammox activity at high concentrations. Therefore, understanding the upper and lower-limit concentrations of each heavy metal required by anammox bacteria is essential for stable operation of the anammox process.

Zhang et al. (2019) reported that a long-term exposure to 1 mg L^−1^ of Zn(II) inhibits the anammox activity. The anammox process is also rapidly inhibited by a mixture of Zn(II), Cu(II), and Ni(II) added at the concentrations of 0.8, 0.07, and 0.04 mg L^−1^, respectively, and probably, the main inhibitor was 0.8 mg L^−1^ of Zn(II) (Gutwínski et al., 2021). In our previous work, inhibitory effects of Zn(II) and Ni(II) were observed on the continuous feeding test at the concentrations of 10 and 5 mg L^−1^, respectively (Kimura and Isaka, 2014). The IC_50_ of Ni(II) was determined as 14.6 mg L^−1^ in a short-term batch test (Wu et al., 2019). These results suggest that the Zn(II) and Ni(II) concentrations above the level of 1–15 mg L^−1^ may affect the anammox activity. Accordingly, the Zn(II) and Ni(II) concentrations should be controlled to be below this inhibition level.

To our knowledge, the heavy-metal concentrations below which anammox metabolism is compromised have not been reported. Of course, the elements that are nonessential for bacterial growth have no effect on anammox activity and their lower limits do not need to be determined. Tests with additive heavy-metal concentrations of 0 mg L^−1^ have been reported but the actual concentrations or quantitative lower limits of heavy metals are unknown. When the synthetic wastewater is tap water or groundwater, heavy metals may be present in the water.

The novelty of this study is that the heavy metals required to maintain the anammox activity were evaluated by limiting each heavy metal in long-term tests. To avoid contamination by heavy metals, ultrapure water was used as the synthetic wastewater and all test systems were installed in a clean booth equipped with an FFU. An ICP–MS analysis confirmed that contamination by limiting elements was minimized in the present study, with levels below 0.001 mg L^−1^. The start-up of the anammox reactor did not require Se(VI) and Ni(II) but required Zn(II) at levels of ≥0.005 mg L^−1^, preferably at 0.020 mg L^−1^. The Zn(II) concentration can be reduced to approximately one-fifth of that of the previously proposed value (van der Star et al., 2007). Meanwhile, Se(VI) and Ni can be omitted from the anammox process or supplied intermittently rather than continuously. As Se(VI) is toxic and its handling is restricted in Japan, its omission from the cultivation of anammox bacteria is important for the management of toxic substances and the safety of researchers.

In this study, the concentration of each heavy metal remained below 0.001 mg L^−1^ but was never completely zero. Heavy-metal concentration can remain at the pg L^−1^ level. Therefore, it cannot be ruled out that the anammox activity was maintained by very low levels (<0.001 mg L^−1^) of heavy metals. In the Zn (II)-limitation tests, anammox activity was retained at 0.005–0.020 mg L^−1^, suggesting that heavy metals at a concentration of 0.005 mg L^−1^ are available in the major enzymes of anammox bacteria. The appropriate concentrations of other elements in anammox reactors should be investigated in the future.

Anammox processes have mainly been introduced to municipal wastewater treatment, in which the shortage of TEs is not problematic because the TE levels are sufficient (Lackner et al., 2014). However, anammox processes are also expected to be applied in industrial wastewater treatment (Tokutomi et al., 2011; Isaka et al., 2017). In industrial wastewaters such as semiconductor and ammonia-plant wastewaters, the levels of heavy metals are insufficient to support anammox activity owing to the use of pure water in the production lines. In this case, heavy metals must be added to the wastewater as chemicals for the biological nitrogen removal process. Minimizing the levels of these elements would considerably reduce the operational cost (i.e., the cost of chemicals). In addition, as the wastewater is ultimately released into the environment, minimizing the amounts of heavy metals is preferred from an environmental-protection viewpoint. For these purposes, clarifying the necessity of adding Se(VI), Zn(II), and Ni(II) and determining the lower-limit concentration of Zn(II) are essential.

To understand the necessity for heavy metals, we must focus on the metal-based enzymes involved in the anammox reaction. Hydrazine synthase (HZS) is a specific enzyme involved in N_2_H_4_ production from NO and NH_4_^+^ in the anammox reaction. As HZS contains Zn (Dietl et al., 2015), we conclude that Zn(II) is essential for the anammox reaction.

Meta 16S rRNA gene sequencing revealed the predominance of *Candidatus* Kuenenia stuttgartiensis and the stability of bacterial community structure in gel carriers under limited concentration of TE. Although a decrease in anammox activity was observed under Zn-limitation condition, anammox activity was restored by the addition of Zn(II). This finding coincided with the slight increase in the relative abundance of anammox bacteria by the addition of Zn(II). Further, (Li et al., 2018). reported that Zn(II) increased the activities of anammox bacteria and the total population of the sludge in an anammox system in an SBR. These results suggest that the change in the anammox activity owing to the limited concentration of TEs may be caused by the change in the number of anammox bacteria. However, the possibility of the change in the cellular activity of anammox bacteria by varying Zn(II) concentrations should be considered. Thus, in the future studies, the effect of TEs on the biological activity of anammox bacteria should be evaluated at the molecular level.

In this study, we investigated whether heavy metals are required as TEs in the anammox process using gel carriers. The gel entrapment system effectively maintains the anammox biomass in the reactor, but the need for TEs on the anammox processes is not conclusive for two reasons. First, the sensitivities of different types of anammox processes must be considered. As the cells were maintained inside the gel carriers, the anammox biomass was less likely to outflow than in other anammox processes and the heavy-metal limitation effect was possibly suppressed. In the future work, similar studies should be conducted in treatment systems using other anammox processes. Second, we should consider the need for TEs in the nitritation process, an important pretreatment process of the anammox process. Some heavy metals that are not required in the anammox process may be required in the nitritation process.

(Van der Star et al., 2007) proposed nine heavy metals, namely, Fe(II), Zn(II), Mn(II), Cu(II), Ni(II), Mo(VI), Co(II), B(Ⅲ), and Se(VI), that ensure stable cultivation of anammox bacteria. Their study has been pivotal in the development of current anammox research. In the present study, we reported only the limitation effects of Se(VI), Zn(II), and Ni(II). The remaining six elements are under investigation, and the results will be reported once the testing is completed.

## Conclusion

Heavy metals inhibit anammox activity at high concentrations but are required in trace amounts for anammox activity, which are not always provided in wastewater treatments. In the present study, the TEs required to start-up and maintain the anammox process in gel carriers were evaluated in a continuous feeding test. The long-term limiting effects of heavy metals present at levels below 0.001 mg L^−1^ were evaluated. In the absence of Se(VI) and Ni(II), the anammox process was started-up without inhibition and the nitrogen removal performance remained stable for more than 2 months. Therefore, Se(VI) and Ni(II) are considered unnecessary for the anammox process; at least, their continuous supply is unnecessary.

By contrast, Zn(II) was required for start-up of the anammox reactor. The nitrogen removal performance was insufficient in the Zn(II)-limited reactor and the anammox activity gradually reduced. In the present system, Zn(II) must be supplied at a concentration of 0.005 mg L^−1^ to maintain anammox activity and at 0.020 mg L^−1^ to recover the anammox activity after deactivation by Zn(II) limitation. Consistent with these findings, Zn(II) is a component of the HZS enzyme that catalyzes N_2_H_4_ production from NO and NH_4_^+^. The Zn(II) concentration required for sustained anammox activity was approximately one-fifth of that proposed in a previous study.

The anammox reaction is expected in future treatments of industrial wastewater. When the Zn(II) concentration in wastewater is insufficient, stable nitrogen removal performance can be obtained by adding Zn(II) at a preferable concentration to the anammox reactor.

## Data Availability

The datasets presented in this study can be found in online repositories. The names of the repository/repositories and accession number(s) can be found below: DDBJ Sequence Read Archive (DRA), DRA013457.

## References

[B1] Caporaso J. G. Kuczynski J. Stombaugh J. Bittinger K. Bushman F. D. Costello E. K. (2010). QIIME Allows Analysis of High-Throughput Community Sequencing Data. Nat. Methods 7, 335–336. 10.1038/nmeth.f.303 20383131 PMC3156573

[B2] Dietl A. Ferousi C. Maalcke W. J. Menzel A. de Vries S. Keltjens J. T. (2015). The Inner Workings of the Hydrazine Synthase Multiprotein Complex. Nature 527, 394–397. 10.1038/nature15517 26479033

[B3] Dimitrova I. Dabrowska A. Ekström S. (2020). Start-up of a Full-Scale Partial Nitritation-Anammox MBBR without Inoculum at Klagshamn WWTP. Water Sci. Technol. 81, 2033–2042. 10.2166/wst.2020.271 32666956

[B4] Fermoso F. G. Bartacek J. Jansen S. Lens P. N. L. (2009). Metal Supplementation to UASB Bioreactors: from Cell-Metal Interactions to Full-Scale Application. Sci. Total Environ. 407, 3652–3667. 10.1016/j.scitotenv.2008.10.043 19091385

[B5] Ferris M. J. Muyzer G. Ward D. M. (1996). Denaturing Gradient Gel Electrophoresis Profiles of 16S rRNA-Defined Populations Inhabiting a Hot spring Microbial Mat Community. Appl. Environ. Microbiol. 62, 340–346. 10.1128/aem.62.2.340-346.1996 8593039 PMC167804

[B6] Fujii T. Sugino H. Rouse J. D. Furukawa K. (2002). Characterization of the Microbial Community in an Anaerobic Ammonium-Oxidizing Biofilm Cultured on a Nonwoven Biomass Carrier. J. Biosci. Bioeng. 94, 412–418. 10.1016/s1389-1723(02)80218-3 16233327

[B7] Furukawa K. Lieu P. K. Tokitoh H. Fujii T. (2006). Development of Single-Stage Nitrogen Removal Using Anammox and Partial Nitritation (SNAP) and its Treatment Performances. Water Sci. Technol. 53, 83–90. 10.2166/wst.2006.175 16749443

[B8] Glass J. B. Orphan V. J. (2012). Trace Metal Requirements for Microbial Enzymes Involved in the Production and Consumption of Methane and Nitrous Oxide. Front. Microbio. 3, 61. 10.3389/fmicb.2012.00061 PMC3282944 22363333

[B9] Gutwínski P. Cema G. Ziembínska-Buczýnska A. Wyszýnska K. Surmacz-Górska J. (2021). Long-term Effect of Heavy Metals Cr(III), Zn(II), Cd(II), Cu(II), Ni(II), Pb(II) on the Anammox Process Performance. J. Water Process. Eng. 39, 101668. 10.1016/j.jwpe.2020.101668

[B10] Isaka K. Date Y. Sumino T. Tsuneda S. (2007). Ammonium Removal Performance of Anaerobic Ammonium-Oxidizing Bacteria Immobilized in Polyethylene Glycol Gel Carrier. Appl. Microbiol. Biotechnol. 76, 1457–1465. 10.1007/s00253-007-1106-6 17653541

[B11] Isaka K. Kimura Y. Matsuura M. Osaka T. Tsuneda S. (2017). First Full-Scale Nitritation-Anammox Plant Using Gel Entrapment Technology for Ammonia Plant Effluent. Biochem. Eng. J. 122, 115–122. 10.1016/j.bej.2017.03.005

[B12] Jis General. (2007). Rules for High Frequency Plasma Mass Spectrometry. Jpn. Stand. Assoc.

[B13] Jis General. (2017). Rules for Ion Chromatography. Jpn. Stand. Assoc.

[B14] Kimura Y. Isaka K. (2014). Evaluation of Inhibitory Effects of Heavy Metals on Anaerobic Ammonium Oxidation (Anammox) by Continuous Feeding Tests. Appl. Microbiol. Biotechnol. 98, 6965–6972. 10.1007/s00253-014-5735-2 24723296

[B15] Lackner S. Gilbert E. M. Vlaeminck S. E. Joss A. Horn H. Van Loosdrecht M. C. M. (2014). Full-scale Partial Nitritation/anammox Experiences - an Application Survey. Water Res. 55, 292–303. 10.1016/j.watres.2014.02.032 24631878

[B16] Li J. Feng L. Qiang Z. Dong H. Wang D. (2018). Enhanced Performance and Kinetics of marine Anammox Bacteria (MAB) Treating Nitrogen-Rich saline Wastewater with Mn(II) and Ni(II) Addition. Bioresour. Tech. 249, 1085–1091. 10.1016/j.biortech.2017.10.101 29137929

[B17] Li Y. Cui N. Xuan K. Xu D. Wang D. Li C. (2021). Start-up Performance and Process Kinetics of a UASB-Anammox Reactor at Low Substrate Concentration. J. Environ. Chem. Eng. 9, 106726. 10.1016/j.jece.2021.106726

[B18] Mulder A. Graaf A. A. Robertson L. A. Kuenen J. G. (1995). Anaerobic Ammonium Oxidation Discovered in a Denitrifying Fluidized Bed Reactor. FEMS Microbiol. Ecol. 16, 177–184. 10.1111/j.1574-6941.1995.tb00281.x

[B19] Peng Z. Lei Y. Liu Y. Wan X. Yang B. Pan X. (2022). Fast Start-Up and Reactivation of Anammox Process Using Polyurethane Sponge. Biochem. Eng. J. 177, 108249. 10.1016/j.bej.2021.108249

[B20] Sindhu L. Niu K. Liu X. Ni S.-Q. Fang X. (2021). Effect of Fe2+ Addition on Anammox Consortia, Nitrogen Removal Performance and Functional Genes Analysis during Start-Up of Anammox Process. J. Water Process Eng. 43, 102251. 10.1016/j.jwpe.2021.102251

[B21] Sliekers A. O. Third K. A. Abma W. Kuenen J. G. Jetten M. S. M. (2003). Canon and Anammox in a Gas-Lift Reactor. FEMS Microbiol. Lett. 218, 339–344. 10.1016/s0378-1097(02)01177-1 12586414

[B22] Strous M. Fuerst J. A. Kramer E. H. M. Logemann S. Muyzer G. Van de Pas-Schoonen K. T. (1999). Missing Lithotroph Identified as New Planctomycete. Nature 400, 446–449. 10.1038/22749 10440372

[B23] Strous M. Heijnen J. J. Kuenen J. G. Jetten M. S. M. (1998). The Sequencing Batch Reactor as a Powerful Tool for the Study of Slowly Growing Anaerobic Ammonium-Oxidizing Microorganisms. Appl. Microbiol. Biotechnol. 50, 589–596. 10.1007/s002530051340

[B24] Strous M. Pelletier E. Mangenot S. Rattei T. Lehner A. Taylor M. W. (2006). Deciphering the Evolution and Metabolism of an Anammox Bacterium from a Community Genome. Nature 440, 790–794. 10.1038/nature04647 16598256

[B25] Tokutomi T. Yamauchi H. Nishimura S. Yoda M. Abma W. (2011). Application of the Nitritation and Anammox Process into Inorganic Nitrogenous Wastewater from Semiconductor Factory. J. Environ. Eng. 137, 146–154. 10.1061/(asce)ee.1943-7870.0000303

[B26] van der Star W. R. L. Abma W. R. Blommers D. Mulder J.-W. Tokutomi T. Strous M. (2007). Startup of Reactors for Anoxic Ammonium Oxidation: Experiences from the First Full-Scale Anammox Reactor in Rotterdam. Water Res. 41, 4149–4163. 10.1016/j.watres.2007.03.044 17583763

[B27] Wu D. Zhang Q. Xia W.-J. Shi Z.-J. Huang B.-C. Fan N.-S. (2019). Effect of Divalent Nickel on the Anammox Process in a UASB Reactor. Chemosphere 226, 934–944. 10.1016/j.chemosphere.2019.03.121 31509923

[B28] Xu J.-J. Zhang Z.-Z. Chen Q.-Q. Ji Z.-Q. Zhu Y.-H. Jin R.-C. (2017). The Short- and Long-Term Effects of Mn2+ on Biogranule-Based Anaerobic Ammonium Oxidation (Anammox). Bioresour. Tech. 241, 750–759. 10.1016/j.biortech.2017.06.014 28628979

[B29] Zhang X. Chen Z. Zhou Y. Ma Y. Ma C. Li Y. (2019). Impacts of the Heavy Metals Cu (II), Zn (II) and Fe (II) on an Anammox System Treating Synthetic Wastewater in Low Ammonia Nitrogen and Low Temperature: Fe (II) Makes a Difference. Sci. Total Environ. 648, 798–804. 10.1016/j.scitotenv.2018.08.206 30138879

