# Supplementary material for: Long-Term Limitation Effects of Se(VI), Zn(II), and Ni(II) on Start-Up of the Anammox Process Using Gel Carrier
Source: Front Bioeng Biotechnol. 2022 Mar 4;10:851617. doi: 10.3389/fbioe.2022.851617 (PMC8931481; doi:10.3389/fbioe.2022.851617)
Supplement: Supplementary file 1 [file Image1.pdf]

Supplementary Material

Figure S1

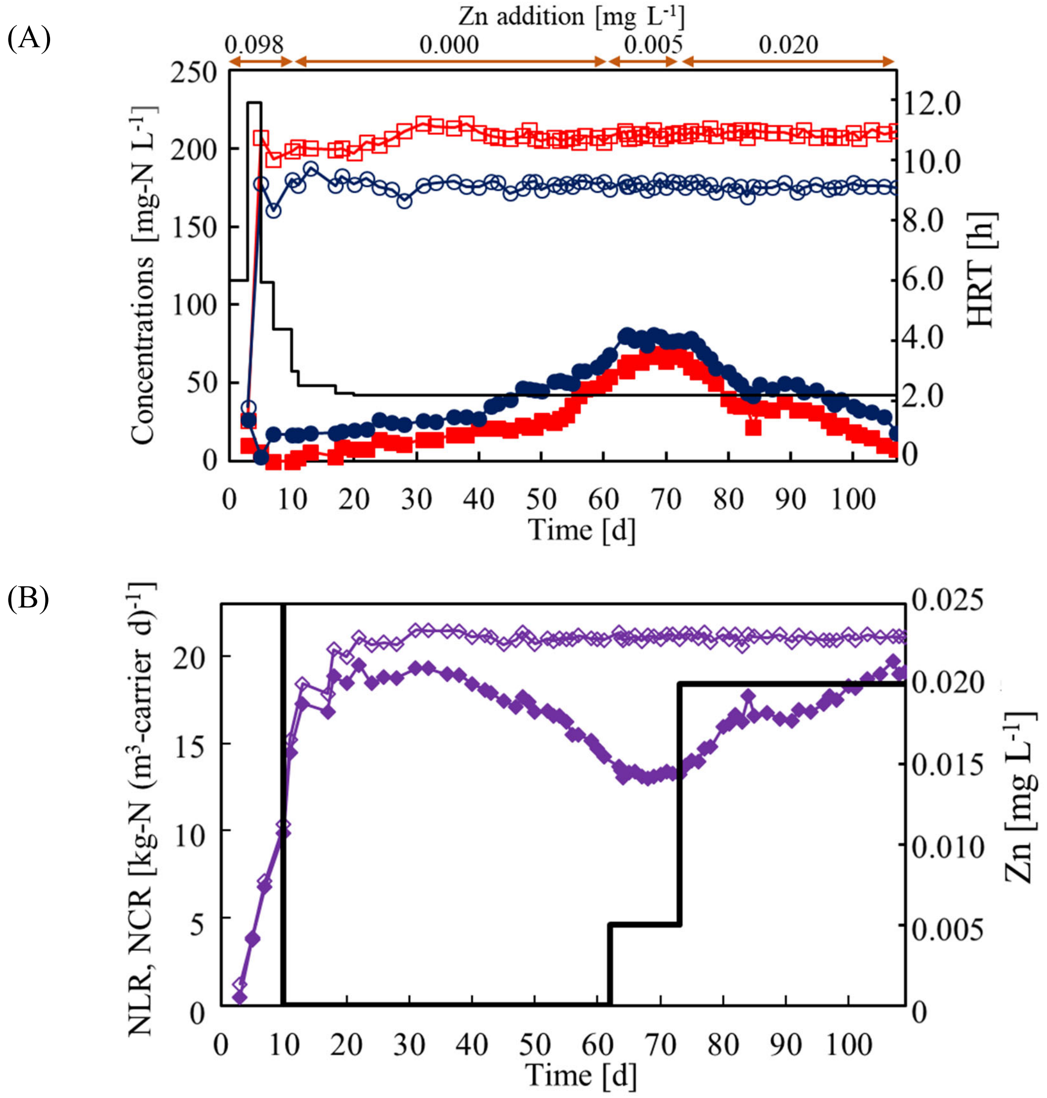

Fig. S1. Zn(II) limitation test on the star-up of the anammox reactor using gel carrier for preliminary test. (A) Influent and effluent nitrogen concentration over time in days. Symbols are as follows: influent ammonium (open circles), influent nitrite (open squares), effluent ammonium (filled circles), effluent nitrite (filled squares), and effluent nitrate (filled triangles). The bar graph shows HRTs. (B) Time courses of nitrogen loading (open diamonds) and nitrogen conversion rate (filled diamonds). Bar graph shows the set value of the influent Zn(VI) on each day.
